# Islet-Specific CTL Cloned from a Type 1 Diabetes Patient Cause Beta-Cell Destruction after Engraftment into HLA-A2 Transgenic NOD/SCID/IL2RG Null Mice

**DOI:** 10.1371/journal.pone.0049213

**Published:** 2012-11-14

**Authors:** Wendy W. J. Unger, Todd Pearson, Joana R. F. Abreu, Sandra Laban, Arno R. van der Slik, Sacha Mulder-van der Kracht, Michel G. D. Kester, Dave V. Serreze, Leonard D. Shultz, Marieke Griffioen, Jan Wouter Drijfhout, Dale L. Greiner, Bart O. Roep

**Affiliations:** 1 Department of Immunohematology and Blood Transfusion, Leiden University Medical Center, Leiden, The Netherlands; 2 Department of Medicine, University of Massachusetts Medical School, Worcester, Massachusetts, United States of America; 3 Department of Hematology, Leiden University Medical Center, Leiden, The Netherlands; 4 The Jackson Laboratory, Bar Harbor, Maine, United States of America; Children’s Hospital Boston/Harvard Medical School, United States of America

## Abstract

Despite increasing evidence that autoreactive CD8 T-cells are involved in both the initiation of type 1 diabetes (T1D) and the destruction of beta-cells, direct evidence for their destructive role *in-vivo* is lacking. To address a destructive role for autoreactive CD8 T-cells in human disease, we assessed the pathogenicity of a CD8 T-cell clone derived from a T1D donor and specific for an HLA-A2-restricted epitope of islet-specific glucose-6-phosphatase catalytic-subunit related protein (IGRP). HLA-A2/IGRP tetramer staining revealed a higher frequency of IGRP-specific CD8 T-cells in the peripheral blood of recent onset human individuals than of healthy donors. IGRP_265–273_-specific CD8 T-cells that were cloned from the peripheral blood of a recent onset T1D individual were shown to secrete IFNγ and Granzyme B after antigen-specific activation and lyse HLA-A2-expressing murine islets *in-vitro*. Lytic capacity was also demonstrated *in-vivo* by specific killing of peptide-pulsed target cells. Using the HLA-A2 NOD-*scid IL2rγ^null^* mouse model, HLA-A2-restricted IGRP-specific CD8 T-cells induced a destructive insulitis. Together, this is the first evidence that human HLA-restricted autoreactive CD8 T-cells target HLA-expressing beta-cells *in-vivo*, demonstrating the translational value of humanized mice to study mechanisms of disease and therapeutic intervention strategies.

## Introduction

Non-obese diabetic (NOD) mice are widely used for studying the pathogenesis and therapy of autoimmune type 1 diabetes (T1D) [1]. Autoreactive CD4 and CD8 T-cells in NOD mice infiltrate and subsequently destroy the insulin-producing beta-cells in pancreatic islets, resulting in dysregulation of blood glucose levels and hyperglycemia [2]; [3]. Although studies using NOD mice have helped to define mechanisms of disease pathogenesis and to identify potential interventional and therapeutic strategies in autoimmune diabetes, there are discrepancies between disease pathogenesis in NOD mice and in humans. For example, while the lymphocytic infiltrate in the NOD pancreas is extensive, insulitis in humans is multi-focal and targets islets scattered throughout a pancreas [1]; [4].

The recruitment of autoreactive CD4 T-cells is required for the initiation of autoreactivity in NOD mice, yet CD8 T-cells are increasingly being recognized as key pathogenic mediators in the destruction of beta-cells and progression of the disease in both diabetic NOD mice and humans [5]–[11]. Deficiency of CD8 T-cells due to the absence of MHC class I in NOD mice renders these mice completely resistant to T1D [6]; [7]. In humans, susceptibility to T1D is strongly associated with the HLA-A2 haplotype, with 60–70% of affected individuals expressing this allele [12]. Transgenic expression of HLA-A2 significantly accelerates disease onset in NOD mice, and is associated with the early appearance of HLA-A2-restricted CD8^+^ T-cells in prediabetic insulitic lesions [13].

The epitopes recognized by autoreactive cytotoxic T-cells are thought to be primarily derived from beta-cell proteins, and the identity of these epitopes in humans remains under active investigation. A known target of autoreactive CD8 T-cells in the NOD mouse is islet-specific glucose-6-phosphatase catalytic-subunit related protein (IGRP) [14]; [15]. Several epitopes from IGRP have been identified using HLA-A2 transgenic (Tg) NOD mice [16], and some of these epitopes are also targeted by CD8 T-cells in T1D individuals [17]; [18]. In addition, the IGRP-specific CD8 T-cells from HLA-A2 Tg NOD mice lyse human HLA-A2^+^ beta-cells *in-vitro*, demonstrating the potential utility of HLA-A2 Tg NOD mice for identifying HLA-restricted islet autoantigens and for investigating the pathogenesis of human T1D [16].

To date, however, direct evidence that human diabetogenic CD8 T-cells target beta-cells *in-vivo* is lacking. The diabetogenic role of CD8 T-cells was suggested by correlation between increased frequencies of islet autoreactive CD8 T-cells in T1D islet allograft recipients that exhibited recurrent islet autoimmunity and loss of graft beta-cell function [11]. Furthermore, islet-autoreactive CD8 T-cells from individuals reactive with preproinsulin selectively killed human islet cells *in-vitro* in a glucose concentration-dependent fashion, suggesting cross-talk between the immune system and pancreatic beta-cells [19]. Very recently, we could demonstrate the presence of IGRP-specific CD8 T cells in insulitis lesions of human T1D patients, which is strong indication for their role in the beta-cell destruction process in humans [20]. To close the critical gap in knowledge of disease mechanisms in T1D in humans, novel preclinical models are needed to investigate the pathogenicity of human autoreactive T-cells *in-vivo*. Recently, NOD-*scid IL2rγ^null^* mice have been developed [21]–[23] that readily engraft with human PBL [24] and are transgenic for HLA-A2 [25]; [26].

We and others have shown that human HLA-A2-restricted CD8 T-cells can recognize the IGRP_265–273_ epitope conserved between mice and humans [18]; [27]. To test the involvement of CD8 T-cells specific for this epitope in beta-cell destruction, we cloned these cells from the peripheral blood of a recent onset T1D individual. We then used the new generation of HLA-A2 Tg immunodeficient humanized mice to assess the pathogenicity of human effector immune cells *in-vitro* and *in-vivo* [28].

## Results

### IGRP_265–263_-specific CD8 T-cells Cloned from the Blood of Type 1 Diabetic Individuals are Effector CTLs

**Figure 1:**
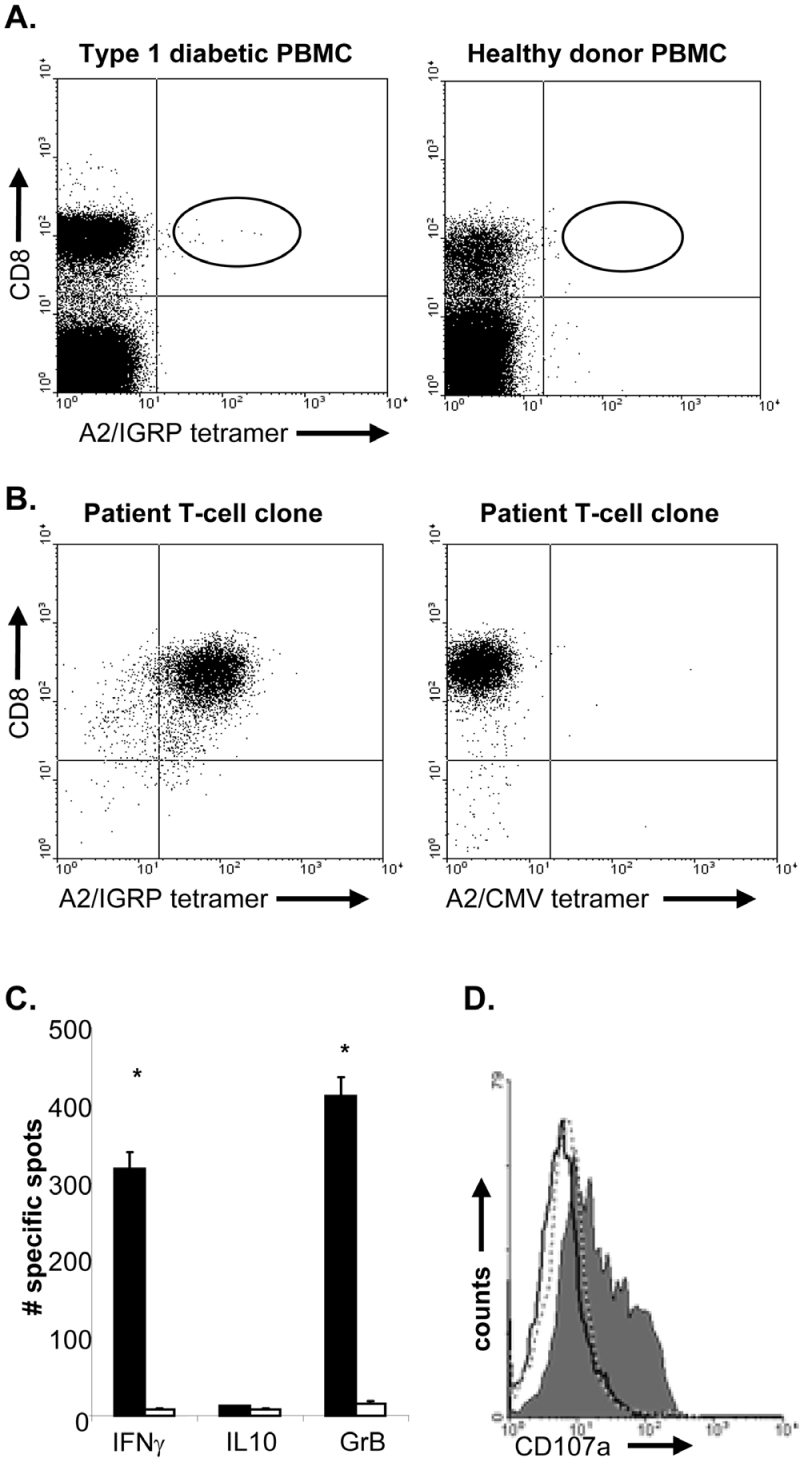
IGRP_265–273_-specific T-cells cloned from the peripheral blood of type 1 diabetic individuals. **A,** PBMCs from a HLA-A*0201^+^ recent onset diabetic patients (left panel) and HLA A*0201^+^ healthy donors (right panel) were incubated with A2/IGRP tetramers, followed by incubation with anti-CD8. CD8/tetramer double positive T cells were only detected in PBMC obtained from type 1 diabetic individuals and were not detected in the blood obtained from healthy controls. **B,** CD8/tetramer double positive T-cells were sorted at one cell per well and clones were picked. IGRP-specific T-clones stained with IGRP specific tetramers were observed in wells derived from type 1 diabetic individuals (left panel). These clones did not bind control HLA-A2 tetramers (right panel). **C,** To assess their cytokine production profile, T-cells were incubated with IGRP peptide-pulsed or control peptide-pulsed HLA-A2 EBV-LCL on anti-IFNγ; anti-GrB and anti-IL10-coated ELISpot plates. Shown is the average number of spots of triplicate wells. Data are representative of 3 independent experiments. * indicates significant difference from controls, *P*<0.01. **D,** IGRP-specific T-cells were incubated with control peptide-pulsed (dashed line) or IGRP peptide-pulsed HLA-A2 EBV-LCL in the presence of anti-CD107a (grey histogram) antibodies. As a control, T-cells were incubated with IGRP peptide-pulsed target cells in the presence of isotype control antibodies (black line) for 5 hours. T-cells were stained for CD8 and expression of CD107a was analyzed on CD8^+^ T-cells using flow cytometry. Results are representative of 2 independent experiments.

IGRP_265–273_ has been identified as an epitope of islet autoreactive CD8 T cells using NOD-*β2m^tm1Unc^ HHD* Tg mice that express a chimeric HLA-A2.1/H-2D^b^ molecule [16]. To assess potential involvement of CD8 T-cells specific for this epitope in islet-cell destruction *in-vivo,* IGRP_265–273_ cells were cloned from the peripheral blood of a recent onset T1D individual. Selective binding of this epitope to HLA-A2 was determined (Figure S1). Using A2/IGRP tetramers and CD8 antibodies, IGRP-specific CD8 T-cells were identified in PBMC of HLA-A2^+^ recent onset individuals but not of healthy controls, confirming previous findings (Figure 1A). Double positive cells were sorted at one cell per well and stimulated using peptide-pulsed APC and feeders. Three weeks later, IGRP-specific CD8 T-cell clones were identified that stained with HLA-A2-specific tetramer but not with an HLA-A2-control tetramer (Figure 1B).

**Figure 2:**
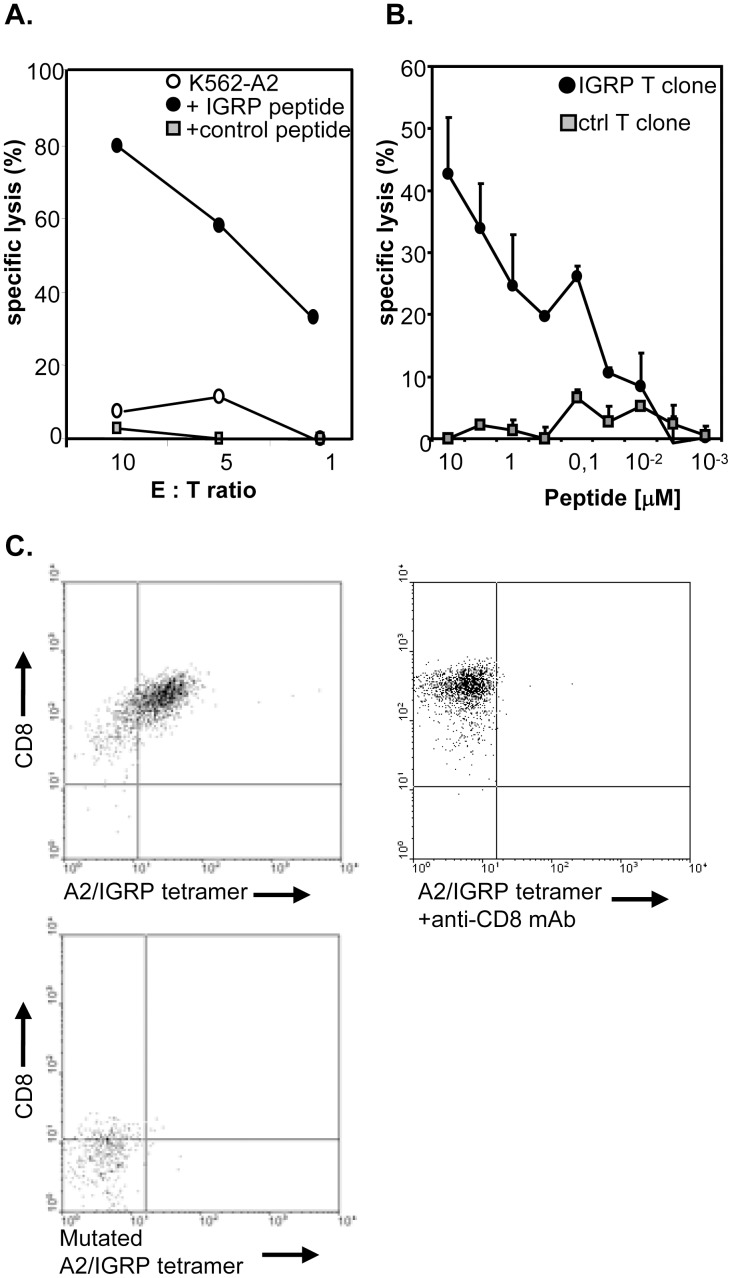
Low-avidity IGRP-specific T-cells exhibit lytic activity *in-vitro*. **A,** Lytic capacity of the IGRP-specific T-cell was examined in a standard 4 h cytotoxicity assay. 3000 ^51^Cr labeled HLA-A2^+^ IGRP- (black dots), control-(grey squares) or non-peptide (white dots) pulsed targets were incubated with the IGRP-specific T-cells at the indicated ratios. **B,** Avidity of the IGRP-specific T-cells was examined by titrating the amount of specific (black dots) or control peptide (grey squares) on peptide-pulsed HLA-A2^+^ target cells. **C,** Tetramer staining in the presence of anti-CD8 antibody. IGRP-specific CTLs were tested 15 days after the last antigenic stimulation. Cells were labelled with A2/IGRP tetramer in the absence (left plot) or presence of an anti-CD8 antibody (1 µg/ml of SK1; BD Biosciences; middle plot), or T-cells were incubated with a mutated IGRP/A2 tetramer (right plot). After tetramer labelling T-cells were stained with anti-CD8 and expression was analysed by flow cytometry. Examples shown are representative of 3 independent experiments.

From the available clones, we selected one for further characterization. Analysis of clone 7 TCR-Vα and -Vβ transcripts using specific primers revealed message for Vα19 and Vβ13 only, which, in combination with VDR3 length variation analysis, confirmed clonality of this T-cell population (Table S1). Clone 7 T-cells (hereafter referred to as IGRP-specific T-cells) expressed markers necessary for strong antigen recognition (CD2 and CD28), as well as the activation- and memory markers HLA-DR and CD45RO (Figure S2). Activation of the IGRP_265–273_-specific T-cells with their cognate antigen resulted in secretion of IFNγ but not IL-10 (Figure 1C). No cytokine production was detected when APC were loaded with a control HLA-A2 binding peptide, demonstrating the antigen specificity of these T-cells (Figure 1C). In addition, IGRP-specific T-cells produced IL-2 and TNFα upon antigen-specific activation, but not IL-4 (data not shown), endorsing these cells with an overt effector phenotype [29]. Furthermore, antigen-specific activation of these CD8 T-cells led to cytotoxic activity as shown by expression of CD107a, which is present in the membrane of cytotoxic granules, production of GrB as well as lysis of peptide-pulsed HLA-A2^+^ target-cells (Figure 1C,D and 2A). The efficacy of the IGRP-specific T-cell clone to recognize the IGRP_265–273_ epitope was demonstrated in peptide-dose titration cytotoxicity assays, indicating that the IGRP-specific T-cell clone had a relatively low avidity for its ligand, since 1 µM peptide was required to attain 50% of the maximal response (Figure 2B). A hallmark of low-avidity T-cells is CD8-dependent binding of peptide/MHC complexes [30]; [31]. Anti-CD8 antibody completely blocked tetramer labeling (Figure 2C). Furthermore, T-cells stained negative with IGRP_265–273_ tetramers of HLA-A2 molecules mutated in their CD8-binding α3 region [32]; [33].

### Lysis of HLA-A2 Expressing Murine Islets *in-vitro*

We next examined the potential of the IGRP-specific T-cell clone to recognize naturally processed and presented IGRP epitope by testing its reactivity with HLA-A2 expressing murine islets. Since dissociated islet-cells exhibit a high degree of spontaneous death that can complicate data interpretation, we used a previously described assay in which intact islets are allowed to adhere and form monolayers before use [16]. We observed highly efficient islet cell killing of NOD-*scid HHD* Tg islets, even at very low effector:target (E:T) ratiós (Table 1), approximately 15% of the NOD-*scid. HHD* Tg islet cells were lysed at an E:T ratio of 30, whereas only 0.6% HLA-A2-negative islets were killed (P<0.01). Lysis of NOD-*scid HHD* islets by IGRP-specific CD8 T-cells was significantly greater than with HLA-A2-restricted tumor-antigen-specific CD8 T-cells (Table 1).

**Table 1:** IGRP-specific T-cells lyse HLA-A2-positive islets *in-vitro.*

specific cytotoxicity (% +/− sem)				
E : T ratio	NOD-scid HHD islets		NOD-scid islets	
	IGRP-T clone	Control T clone	IGRP-T clone	Control T clone
30∶1	14.7±3.2* ^$^	5.6±0.4	0.6±3.3	1.1±4.3
10∶1	9.8±2.0*		0.3±3.3	

Data represent the percent specific cytotoxicity against the indicated targets by IGRP_265–273_-specific CD8 T cells. HLA-A2-restricted, tumor-antigen-specific CD8 T-cells were used as a control. ^51^Cr release from islets cultured in medium alone (*i.e*. spontaneous release) for each target was measured in 9 independent wells to calculate specific cytotoxicity as described in Materials and Methods. Data are representative of 3 independent experiments.

* indicates a significant difference (p<0.01) in cytotoxicity against NOD-*scid* islets tested at the same E:T ratio; ^$^ indicates a significant difference (p<0.05) in cytotoxicity against NOD-*scid HHD* islets between IGRP-specific CD8 T-cells and control T-cells.

### Human IGRP-specific CD8 T-cells Lyse Peptide-pulsed Target Cells *in-vivo*

To determine the cytotoxic activity of IGRP-specific CD8 T-cells *in-vivo,* we adapted a murine *in-vivo* cytotoxicity assay that is widely used to demonstrate cytotoxic actions of primed effector/memory CD8 T-cells [34]; [35]. HLA-A2^+^ target cells (PBMC) were labeled with different concentrations of CFSE, pulsed with either IGRP_265–273_ or irrelevant peptide, mixed and injected into NOD-*scid IL2rγ^null^* mice that had received an intrasplenic injection of IGRP-specific T-cells 24 h earlier. Mice that had not been injected with the human T-cell clone were used as a negative control. IGRP-specific T-cells lysed IGRP-pulsed target cells *in-vivo*, as demonstrated by the specific disappearance of the CFSE^hi^-labeled target cells pulsed with IGRP-peptide (Table 2). In contrast, the number of CFSE^lo^-labeled targets pulsed with control peptide remained unchanged, even in the presence of the IGRP-specific T-cells.

**Table 2:** *In-vivo* lysis of peptide-pulsed HLA-A2 cell targets by IGRP-specific CD8 T-cells.

	Lysis of peptide-pulsed targets (% +/− sem)		Lysis of control targets(% +/− sem)	
Experiment 1		13.7±2.1*	2.2±0.92	
Experiment 2		21.85±3.1*	3.24±3.01	

Data represent the percentage of specific cytotoxicity against IGRP-peptide pulsed HLA-A2 EBV-LCL by IGRP-specific CD8 T-cells in two independent experiments. 4×10^6^ IGRP-specific T cells were injected intrasplenically. One day later, mice received an i.v. injection containing of a 1∶1 mixture of specific peptide-pulsed CFSE^hi^ target cells and control peptide-pulsed CFSE^lo^ target cells. At 20 hr, the ratio of CFSE^hi^ and CFSE^lo^ cells in the spleens was analyzed by flow cytometry.

* indicates a significant difference (p<0.05) in cytotoxicity against control targets. In each experiment, 4 mice per group were used.

### Intra-pancreatic Injection of IGRP-specific T-cells into NOD-*scid IL2rγ^null^ HHD* Mice Leads to Infiltration and Islet Disruption

We next investigated whether human IGRP-specific CD8 T-cells can target and destroy HLA-A2 pancreatic beta-cells *in-vivo*. The expression of both CD62L and the chemokine receptors CXCR3 and CCR4 (Figure S2) indicates that the IGRP-specific T-cells express adhesion markers required to migrate to secondary lymphoid tissue and inflamed pancreatic tissue [36]; [37]. Since NOD-*scid IL2rγ^null^ HHD* mice are devoid of cytokines necessary for homeostatic T-cell proliferation, we hypothesized that the survival of the human CD8 T-cell clone would be facilitated by injecting human PBMC from non-diabetic donors 48 h earlier. This time span would be sufficient for the PBMC to expand in the lymphopenic environment and produce the necessary cytokines that may be required for survival of cloned CD8 T-cell in the absence of CD4 T-cell help. As a control, a diabetes-unrelated CD8 T-cell clone was injected intrapancreatically. Additional control mice received a sham-injection.

**Figure 3:**
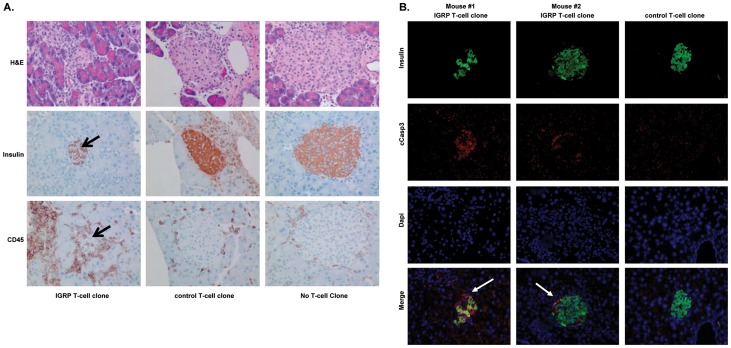
IGRP-specific T-cells are able to infiltrate and destroy beta-cells following intra-pancreatic injection into NOD-*scidIL2rγ^null^ HHD* mice. NOD-*scid IL2rγ^null^ HHD* recipient mice were injected i.v. with 20×10^6^ PBMC from an HLA-A*0201^+^ healthy donor. Two days later, these mice were injected intra-pancreatically with either 5×10^6^ IGRP-specific T-cells (left), 5×10^6^ control T-cells (middle) or were sham injected (right). Four weeks later, the pancreata were isolated and histologically examined. A, Sections of recipients of IGRP-specific or control T-cells, or of those receiving a sham injection were stained with H&E (upper panel) to visualize the histological integrity of the islets or stained for insulin (middle panel) to identify the beta cells or human CD45 (lower panel) to visualize the human T-cells. Similar data were obtained when staining sections for human CD8. B, Pancreatic sections were stained for insulin, cCaspase-3 to detect apoptotic cells and DAPI to identify nuclei. Individual fluorescence as well as an overlay is presented. Shown are representative examples of 5 mice per group.

No changes in blood glucose levels were detected following injection of either IGRP-specific or control T-cell clones (data not shown). Four weeks after adoptive transfer of the human T-cell clones the pancreas was recovered and analyzed by histology for islet integrity and presence of the IGRP-specific CD8 T-cells. Both human IGRP-specific and control CD8 T-cells were present at detectable levels four weeks after injection (Figure 3A). However, their location differed greatly. Pancreata of recipient mice of IGRP-specific T-cells predominantly elicited intra-islet infiltration (Figure 3A). In contrast, control CD8 T-cells remained at the perivascular sites. Mice receiving a sham injection showed no infiltration of islets. Notably, insulin staining of pancreatic sections revealed that the islets were disrupted only in mice injected with the IGRP-specific T-clone, but not in those receiving the control CD8 T-cells or a sham injection (Figure 3A). The number of islets infiltrated with T-cells was significantly greater in mice injected with the IGRP T-clone, 10/49 (20%) than mice injected with the control T-clone, where none of 20 investigated islets showed infiltrating cells, although T-cells were occasionally found around the islets. (p = 0.013; two-tailed Fisher exact test). Moreover, absence of insulin staining was accompanied by positivity for cCaspase-3, pointing to beta-cell destruction by IGRP-specific T-cells (Figure 3B). Pancreata of mice that received control CD8 T-cells were negative for cCaspase-3 staining.

## Discussion

This study provides the first evidence that autoreactive CD8 T-cells cloned from a T1D individual can participate in islet cell destruction *in-vivo* using a novel humanized mouse model. IGRP_265–273_-specific CD8 T-cells producing IFNγ and expressing CD107a upon antigen-specific activation are capable of lysing murine HLA-A2 expressing islets *in-vitro*. *In-vivo*, these T-cells lysed target cells pulsed with islet peptide. Following intra-pancreatic injection into HLA-A2 transgenic NOD-*scid IL2rγ^null^ HHD* mice, IGRP-specific CTLs localized to the islets and caused beta-cell destruction. However, no changes in blood glucose-levels were observed. No islet cell destruction was detected when mice were injected with equal numbers of control HLA-A2-restricted cloned CD8 T-cells.

The assumption that IGRP-specific CD8 T-cells possess diabetogenic potential *in-vivo* was based on the observation that these cloned T-cells were highly cytotoxic for HLA-A2 expressing islets *in-vitro.* However, the extent of lysis observed was low in comparison to lysis of peptide-pulsed EBV-LCL. This difference might be explained by lower expression of HLA-A2 molecule (HHD) on murine islets compared with HLA-A2 levels on human cells, which consequently results in decreased presentation of the IGRP epitope. In addition, the IGRP-specific T-cells possess low-avidity T-cell receptors as revealed by low staining with specific tetramer and CD8 dependency of tetramer staining. This may be inherent to autoreactive T-cells, since these should have been eliminated during thymic education due to high-avidity interactions with their cognate self-antigen. Thus, besides weak recognition of the epitope by the low-avidity TCR of the IGRP-specific T-cells, these observations are also consistent with low amount of IGRP epitope presentation on the surface of the islets.

Using an adapted *in-vivo* cytotoxicity assay, we obtained evidence that IGRP T-cells are capable of lysing targets *in-vivo*. Mobilization of effector CD8 T-cells to infected tissue requires CD4 T-help [38], however, CD4 T-cells only provide this help when specifically activated by their cognate antigen at this site [39]. Given that human IGRP-specific CD4 T-cells were not available, we opted to inject the IGRP-specific CD8 T-cells intra-pancreatically and to provide the optimal cytokine milieu for survival of the transferred T-cells by systemic injection of HLA-A2^+^ PBMC from non-diabetic donors prior,for generation of the necessary cytokines (*i.e.* IL2, IL7, IL15) needed for T-cell homeostasis.

Immunohistochemical analysis of the pancreata four weeks after injection of the human CD8 T-cells revealed clear differences in localization between the IGRP-specific T-cells and control T-cells. IGRP-specific CTLs infiltrated into the islets, whereas control T-cells were retained in the peri-vascular areas. Our observation confirms previous findings that islet antigen expression is a key factor in governing the ability of the autoantigen-specific T-cells to accumulate in the islets [36]; [40]; [41]. Interestingly, both CD4 and CD8 T-cells migrating into pancreatic tissue expressed the chemokine receptor CXCR3, while distressed beta-cells produce its ligand CXCL-10 (IP-10) [42]. This feature may present a master switch for migration of islet autoreactive T-cells to the beta-cell environment, as suggested by preclinical studies [43]. Some human leukocytes were noted in the exocrine pancreatic tissue in both mice injected with IGRP-specific T-cells and control T-cells, which may be partly due to their intrapancreatic injection causing tissue damage and chemokine production leading to inflammatory infiltrates. The CD45 staining will target the injected T-clones as well as co-injected PBMC. Yet, since the same PBMC donor was used for all three T cells clones, the insulitis lesions could be attributed to the particular humans T-cell clones injected, rather than the PBMC that were not as feeders of the CD8 T cells.

Injection of the IGRP-specific T-cells resulted in a destructive inflammation, but not when a high number of cells from a control HLA-A2-restricted CD8 T-cell clone were injected. Since no changes in blood glucose-levels of the recipient mice were observed upon injection of the IGRP-specific T-cells in spite of clear islet cell destruction, we speculate that adoptive transfer of islet-specific CD4 T-cells may be required for propagation and sustained CD8 T-cell expansion and cytotoxic activity [44]. A recent study employing the same HLA-A2 NOD-*scidIL2rγ^null^* mouse model showed that high frequencies of CD4 T-cells were present when mice were engrafted with PBMC from a T1D patient [45]. This was paralleled by the presence of diabetogenic epitope-specific CD8 T-cells. However, in this study no islet cell destruction was demonstrated, which may be due to lower frequency of islet-specific T-cells in the pancreas compared to our study. Yet, the fact that IGRP_265–273_-reactive CD8 T-cells were present among the islet-specific CD8 T-cells, as well as the recent finding that specific targeting of IGRP-reactive CD8 T-cells effectively inhibited diabetes development in NOD.β2m^null^.HHD mice [46] corroborate our current findings that this epitope is pathogenic in human disease.

T-cell autoreactivity may require more than 4 weeks for complete beta cell destruction, and require CD4 T-cells acting in concert. Indeed, the limited survival of CD8 T-cell clones in mice in the absence of CD4 T-cells underscores this notion. Mouse CD4 T-cells specific for a low-affinity insulin-derived peptide required 20 weeks before hyperglycemia developed in 50% of recipient mice [47]. Despite the great value of immunocompetent NOD mice, there are discrepancies between the etiopathogenesis of T1D in NOD mice versus humans. These differences include the pattern of insulitis. The intra-islet infiltration pattern of human islet-specific T-cells and islet destruction observed in our study closely resembles the pathogenesis found in human inflamed pancreas [4]; [20] rather than inflammatory lesion typically observed in NOD mice [1]; [4]. The relevance of islet autoreactive T-cells circulating in the blood in the pathogenesis of insulitis in humans remains unresolved. Nonetheless, our current study on human peripheral blood CD8 T-cell clones, as well as our earlier studies on homing of human anti-islet CD4 T-cells clones to pancreatic tissue, imply that circulating human T-cells can contribute to insulitis. Recent studies on islet autoreactivity in an explanted human pancreas graft indicated that circulating islet-antigen autoreactive CD8 T-cells in peripheral blood were also present in pancreatic inflammatory lesions, albeit at a lower frequency. Moreover, additional islet autoreactivities that were below detection levels in the circulation could be detected in the inflamed pancreas [48].

*In conclusion, our data provide first evidence that human autoreactive CD8 T-cells have the potential to target HLA-A2-matched beta-cells in-vivo, demonstrating the translational value of humanized mice to study mechanisms of disease and interventional strategies.*

## Materials and Methods

### Mice

NOD.Cg-*Prkdc^scid^ Il2rg^tm1Wjl^*/Sz (abbreviated as NOD-*scid IL2rγ^null^*) [21], NOD.Cg-*Prkdc^scid^* (abbreviated as NOD-*scid*) [49] and NOD.Cg-*Prkdc^scid^* Tg(HLA-A/H2-D/B2M)1Dvs*/*Dvs (abbreviated as NOD-*scid HHD*) [16] mice were obtained from The Jackson Laboratory (Bar Harbor, ME). NOD.Cg-*Prkdc^scid^ Il2rg^tm1Wjl^ Tg(HLA-A/H2-D/B2M)1Dvs*/DvsSz (abbreviated as NOD-*scid IL2rγ^null^ HHD)* were generated by crossing NOD.Cg-*Prkdc^scid^ Il2rg^tm1Wjl^*/Sz mice with NOD.Cg-*Prkdc^scid^ Tg(HLA-A/H2-D/B2M)1Dvs/*Dvs and selecting for homozygosity of the *Il2rγ^null^* mutation and presence of the HHD transgene. HHD transgenics were maintained in a hemizygous fashion and evaluated for presence of the transgene by PCR or flow cytometry on peripheral blood before use in experiments. Mice at The University of Massachusetts Medical School and at The Jackson Laboratory were housed in a specific pathogen free facility in microisolator cages, and given autoclaved food and maintained on acidified autoclaved water and sulfamethoxazole/trimethoprim medicated water (Goldline Laboratories, Ft. Lauderdale, FL), provided on alternate weeks. All animal use was in accordance with the guidelines of the Institutional Animal Care and Use Committee (IACUC) of the University of Massachusetts Medical School and The Jackson Laboratory and conformed to the recommendations in the Guide for the Care and Use of Laboratory Animals.

### Peptide Synthesis and Generation of HLA-A2-peptide Tetramers

Peptides were synthesized using Fmoc amino acids and PyBop/NMM chemistry. Synthetic peptides were analyzed by reversed phase HPLC (purity was at least 85%) and Maldi-Tof mass spectrometry (expected masses were confirmed). Tetrameric HLA-A2-peptide complexes were prepared as previously described [50].

### Cloning of IGRP_265–273_-specific CD8 T-cells

Peripheral blood mononuclear cells (PBMCs) from a HLA-A*0201^+^ recent onset diabetic patient were incubated with A2/IGRP tetramer on ice. One hour later, cells were incubated with anti-CD8 for 20 min. A2/IGRP tetramer CD8 double-positive T-cells were sorted and seeded at one cell per well in 96-well plates, each well containing 1×10^5^ irradiated (50 Gy) allogeneic PBMCs, 5×10^3^ irradiated (100 Gy) IGRP_265–273_ peptide-pulsed HLA-A2-expressing EBV-LCL in IMDM supplemented with 10% HS, 0.5% LeucoA, 0.1 ng/ml rh-IL12, 10 ng/ml rh-IL7, 25 U/ml rh-IL2 and 5 ng/ml rh-IL15. T-cell clones were isolated and restimulated every 2–3 weeks as described above. Every 3–4 days fresh media containing 5 ng/ml IL-15 and 25 U/ml IL-2 were added. From the panel of IGRP-reactive CD8 T-cell clones, we selected one T-cell clone, termed clone 7 for further characterization.

### ELISpot Assays

To determine secretion of IFNγ, Granzyme B (GrB), and IL-10, 2×10^4^ clone 7 cells were incubated with HLA-A2-expressing EBV-LCL pulsed with an IGRP-specific or a control peptide on anti-cytokine (IFNγ, GrB, and IL-10) antibody-precoated ELISA plates and after o/n culture in IMDM supplemented with 1% HS_,_ plates were developed according to the manufacturer’s protocol (U-CyTech, Utrecht, the Netherlands). Results are expressed as means ± SD of triplicate wells.

### Analysis of CD107a Expression

Expression of CD107a on clone 7 cells was examined as described by Betts et al [51].

### Analysis of TCR-Vα and Vβ Repertoire by RT-PCR and Sequence Analysis

Total RNA was isolated using RNA BEE (Tel-Test, Friendswood, TX). Random-primed cDNA was synthesized according to manufacturer’s instructions (Roche, Indianapolis, IN). PCR amplification of cDNA was performed with Cα or Cß reverse primer (Cα: TgTgggAgATCTCTgCTTCTg,sequence Cß: TCCTTCCCATTCACCCACCAgCTCAgCTC in combination with a specific forward primer for Vα or Vß TCR ([48] for primer sequences). Control PCR amplifications for β-actin were performed with each sample to confirm cDNA integrity. Aliquots of each reaction were run on agarose gel prestained with ethidium bromide, in order to compare amplicon intensities between reactions. Appropriate dilutions were performed prior to analysis when necessary. To establish whether clone 7 cells were monoclonal, PCR products were subjected to nucleotide sequence analysis using ALFexpress sequencer (Pharmacia-Biotech, Sweden).

### Cytotoxicity Assays

Standard ^51^Cr release assays were performed using HLA-A2^+^ EBV-LCL as target cells. Target cells were labeled with ^51^Cr (100 µCi, Perkin Elmer, Billerica, MA) in the absence or presence of 1 ug/ml specific or control peptide for 1 h at 37°C. After extensive washing, cells were suspended in IMDM/10% HS and 3000 labeled target cells were co-incubated at effector to target (E:T) ratios of 20∶1, 5∶1 and 1∶1 in 96 well round-bottom plates in triplicate for 4 h at 37°C. Controls included target cells incubated in medium alone for spontaneous release and target cells in 5% (v/v) Triton X-100 (Sigma, St. Louis, MO) in PBS for maximum release. Radioactivity was measured by a Wallac Wizard 1470 Automatic Gamma Counter. The percentage of cytotoxic activity was calculated using the following formula: % specific lysis = (sample cpm–spontaneous cpm)/(maximal cpm–spontaneous cpm)×100%.

### Cytotoxicity Assays Using Murine Islets as Targets

Cytotoxicity assays using intact mouse islets as targets were performed as described previously [16]. Briefly, NOD-*scid HHD* and NOD-*scid* pancreatic islets (10 islets/well) were allowed to adhere in 96-well plates during a 10-day incubation at 37°C. Adherent islets were labeled with 5µCi/well of ^51^Cr for 3 h at 37°C. Islets were washed and overlaid with 100 µl of medium containing various numbers of cultured clone 7 cells. For establishing E:T ratios, each islet was assumed to contain 750 cells. A minimum of three wells were established for each E:T ratio. Spontaneous release controls consisted of nine wells of labeled islets from each donor cultured in the absence of T-cells. Following a 20 h incubation at 37°C, the radioactivity in two fractions from each well was measured. The first fraction was the culture supernatant, the second was obtained by solubilizing the remaining islets in 200 µl of 2% SDS. The percentage of ^51^Cr release for each well was calculated by the formula [(supernatant cpm)/(supernatant cpm + SDS lysate cpm)] x 100%. This allows normalization for variation in the sizes of individual islets, which could result in differences in the total levels of ^51^Cr incorporation in each well. In turn, percent-specific cytotoxicity was calculated by subtracting the percent ^51^Cr release from islets cultured in medium alone (i.e., spontaneous release) from the release by each well of islets cultured with a given number of T-cells.

### *In-vivo* Cytotoxicity

To examine the cytotoxic potential of clone 7 cells *in-vivo*, an established *in-vivo* cytotoxicity assay was used with modifications [34]; [35]. PBMC from HLA-A*0201^+^ healthy donors were labeled with either 2 µM or 0.2 µM CFSE (Sigma; CFSE^hi^ and CFSE^lo^ cells, respectively) for 15 minutes at 37°C. CFSE^hi^ cells were pulsed with 3 ug/ml IGRP_265–273_ peptide for 45 minutes at 37°C. CFSE^lo^ cells were pulsed with a control peptide. Both cell populations were washed, combined at equal ratios and 5×10^6^ cells of each population were intravenously injected into NOD-*scidIL2rγ^null^* recipient mice via the tail vein. Recipient mice received intrasplenic injections of indicated numbers of clone 7 IGRP-specific CD8 T cells or control T-cells 24 h earlier. Spleens from recipient mice were harvested 20 hours after target cell injection, and survival of each transferred population was assessed by flow cytometry. A minimum of 1×10^3^ CFSE^+^ events were collected from each sample. Specific cytolytic activity was calculated using the following equation: [1– [(CFSE^hi^/CFSE^lo^ in IGRP-T-cell recipients)/(CFSE^hi^/CFSE^lo^ in control T-cell recipients)]×100.

### Adoptive Transfer Experiments

NOD-*scid IL2rγ^null^ HHD* recipient mice were injected i.v. with 20×10^6^ PBMC from a HLA-A*0201^+^ healthy donor. Two days later, mice were injected intra-pancreatically with 5×10^6^ clone 7 cells, 5×10^6^ control tumor-antigen specific T-cells (PRAME-specific T-cell clone) or sham injected. Blood glucose levels were monitored twice weekly. After 4 weeks, pancreata were recovered for histological analysis. Paraffin-embedded tissues were sectioned at 4 µm and stained with hematoxylin and eosin (H&E) or used for immunohistochemistry. Immune-stained tissues utilized antibodies with specificity for insulin, human CD45 (DAKO, Carpinteria, CA), and employed a standard avidin–biotin complex methodology [52]. The sections were developed using 3-diaminobenzidine and counterstained with hematoxylin for light microscopic visualization. Alternatively, sections were incubated with guinea pig anti-insulin that was visualized by staining with anti-guinea pig IgG conjugated toAlexa-488 and rabbit anti-Caspase-3 antibody that was visualized by staining with anti-rabbit IgG conjugated to Alexa-594 (Invitrogen). Nuclei were visualized with DAPI and the stain sections were analyzed using immunofluorescence microscopy. Control protocols included omission of primary antibodies and the use of species-matched nonbinding control antibodies.

### Statistical Analysis

Experimental groups were compared to controls using student’s *t*-test. P<0.05 was considered significant.

## Supporting Information

Figure S1 **IGRP_265–273_ specifically binds to HLA-A2.** Peptide binding to HLA-A1, -A2, -A3 and –B8 was assessed using a competition based binding assay [11]. The percentage of fluorescent-labeled reference peptide bound to a specific HLA-allele at different concentrations of unlabeled IGRP_265–273_ peptide is shown. The IC_50_ value defines the concentration of IGRP_265–273_ peptide needed to inhibit 50% binding of the fluorescent-labeled reference peptide. IC_50_ value HLA-A2∶63 nM; IC_50_ value HLA-A1, HLA-A3, HLA-B8∶12590 nM.
(TIF)

Figure S2 **IGRP-specific T-cells express adhesion molecules important for migration into inflamed pancreas and secondary lymphoid tissue.** IGRP-specific T-cells were stained with antibodies against TCRαβ, CD2, CD28, HLA-DR, CD45RA, CD45RO, CXCR3, CCR4, CCR5, CCR7, CCR7 and CD62L and analyzed by flow cytometry. Dashed lines represent isotype antibody staining, solid lines represent specific antibody staining. These results are representation of 3 experiments.
(TIF)

Table S1 **Clone 7 IGRP/A2 binding T-cells cloned from a type 1 diabetic individual are clonal.** Clonality of IGRP-specific clone 7 T-cells was determined by examining expression of TCR Vα and TCR Vβ chains. Specific PCR with Cα or Cß reverse primer in combination with a specific forward primer for Vα or Vß TCR revealed expression of Vβ13c and Vα19 transcripts in clone 7 T-cells. Control PCR amplifications for β-actin were performed for each sample to confirm cDNA integrity. Total RNA was isolated using RNA BEE (Tel-Test, Friendswood, TX). Random-primed cDNA was synthesized according to manufacturer’s instructions (Roche, Indianapolis, IN). PCR amplification of cDNA was performed with Cα or Cß reverse primer (Cα: TgTgggAg-ATCTCTgCTTCTg, sequence Cß: TCCTTCCCATTCACCCACCAgCTCAgCTC in combination with a specific forward primer for Vα or Vß TCR ([48] for primer sequences). Control PCR amplifications for β-actin were performed with each sample to confirm cDNA integrity. Aliquots of each reaction were run on agarose gel prestained with ethidium bromide, in order to compare amplicon intensities between reactions. Appropriate dilutions were performed prior to analysis when necessary. To establish whether clone 7 cells were monoclonal, PCR products were subjected to nucleotide sequence analysis using ALFexpress sequencer (Pharmacia-Biotech, Sweden).
(TIF)
