# Supplementary figures and images for: Islet-Specific CTL Cloned from a Type 1 Diabetes Patient Cause Beta-Cell Destruction after Engraftment into HLA-A2 Transgenic NOD/SCID/IL2RG Null Mice
Source: PLoS One. 2012 Nov 14;7(11):e49213. doi: 10.1371/journal.pone.0049213 (PMC3498321; doi:10.1371/journal.pone.0049213)

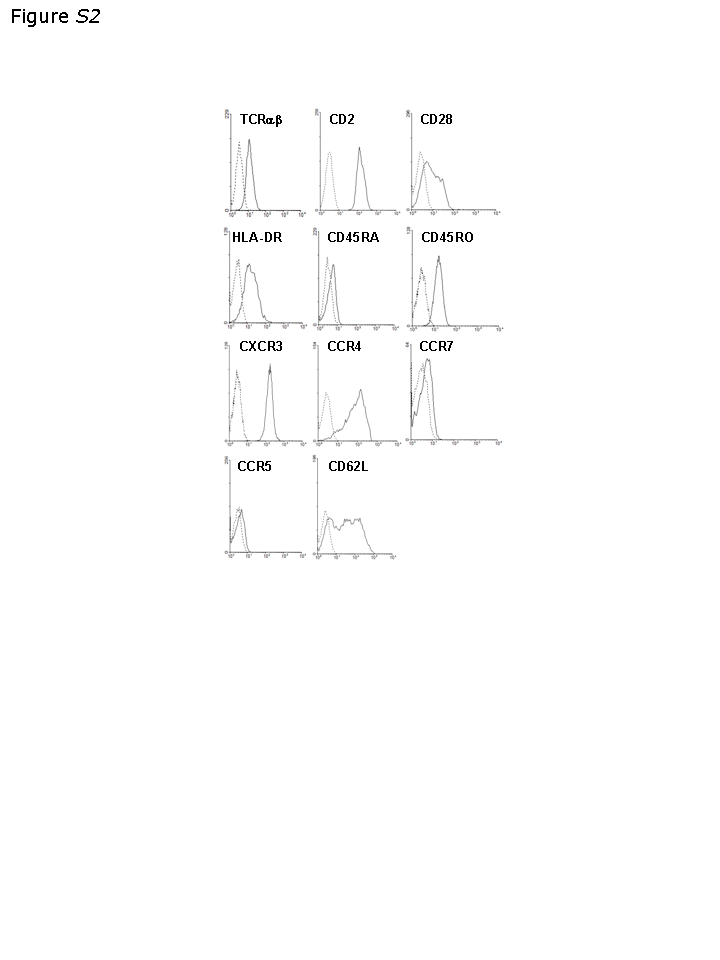

Supplement: Figure S2 — IGRP-specific T-cells express adhesion molecules important for migration into inflamed pancreas and secondary lymphoid tissue. IGRP-specific T-cells were stained with antibodies against TCRαβ, CD2, CD28, HLA-DR, CD45RA, CD45RO, CXCR3, CCR4, CCR5, CCR7, CCR7 and CD62L and analyzed by flow cytometry. Dashed lines represent isotype antibody staining, solid lines represent specific antibody staining. These results are representation of 3 experiments. (TIF) [file pone.0049213.s002.tif]
